# The links between adolescent health and education: pathways, impacts and economic modelling

**DOI:** 10.1093/heapol/czag077

**Published:** 2026-09-30

**Authors:** John Symons, Bruce Rasmussen, Gareth Jones, Hafsah Muheed, Bhavya Nandini, Kenisha Arora, Kim Sweeny, Anshu Mohan, Mily Johana Ramírez Ruiz, Peter Sheehan

**Affiliations:** Victoria Institute of Strategic Economic Studies, Victoria University, PO Box 14428, Melbourne, Victoria 8001, Australia; Victoria Institute of Strategic Economic Studies, Victoria University, PO Box 14428, Melbourne, Victoria 8001, Australia; Partnership for Maternal, Newborn and Child Health, 20 Avenue Appia, Geneva, Switzerland; Independent Scholar; Partnership for Maternal, Newborn and Child Health, 20 Avenue Appia, Geneva, Switzerland; Toronto Metropolitan University, School of Medicine, 150 Central Park Drive, Brampton, Ontario, L6T 1B4, Canada; Victoria Institute of Strategic Economic Studies, Victoria University, PO Box 14428, Melbourne, Victoria 8001, Australia; World Health Organization South-East Asia Regional Office, Family and Gender Health, World Health House, Indraprastha Estate, Mahatma Gandhi Marg, New Delhi 110 002, India; Ministry of Health and Social Protection, Directorate of Promotion and Prevention, Carrera 13 No. 32–76, Teusaquillo, Bogotá 111321, Colombia; Partnership for Maternal, Newborn and Child Health, 20 Avenue Appia, Geneva, Switzerland

**Keywords:** health and education, human capital, adolescent well-being, economic modelling, low- and middle-income countries, benefit-cost ratio (BCR)

## Abstract

Health and education are interdependent pillars of human capital development, yet they are often treated in isolation within policy and economic models. This study presents a conceptual and empirical framework that integrates health and education interventions to assess their joint impact on educational attainment, population health, productivity, and economic development. Using a human capital approach, the study models the effects of four school-based health interventions—nutrition, deworming, water, sanitation, and hygiene (WASH), and mental health programmes—on education outcomes in 75 low- and middle-income countries. It also quantifies the long-term health benefits from reduced adolescent pregnancies, due to increased years of schooling, in the form of lower under-five and adult mortality. The immediate health benefits for adolescents of these school-based programmes are not modelled. Using an updated version of an education model combined with an employment benefits model, the study estimates the costs and returns of investing in these interventions from 2025 to 2050. Results show the interventions alone yield over 422 million additional school completions, with economic benefits exceeding US$15.3 trillion and a benefit-cost ratio (BCR) of 7.0. When health benefits of education in the form of reduced mortality are included, benefits rise to over US$25.2 trillion, with a BCR of 11.5. These findings demonstrate the powerful bidirectional relationship between health and education and support the case for integrated investments. The study also outlines methodological advancements in modelling the combined impact of education and health, while acknowledging limitations such as the focus on market-based outcomes and assumptions of uniform intervention effectiveness. Future research should include immediate economic benefits of health interventions, broader dimensions of human development, including equity, institutional quality, and social capital. The findings offer compelling evidence for policymakers to prioritize coordinated, cross-sectoral strategies that yield high returns in both education and health.

Key messages

- Health and education are deeply interconnected, with improvements in one domain significantly enhancing outcomes in the other across the life course.
- Targeted school-based health interventions—such as nutrition, deworming, WASH and mental health programmes—can substantially increase school completions and educational attainment.
- Modelling across 75 low- and middle-income countries shows that integrated health-education investments yield high economic returns, with benefit-cost ratios exceeding 11.
- Coordinated policy approaches that treat health and education as mutually reinforcing components of human capital can deliver more equitable and sustainable development outcomes.

## Introduction

The interdependence between health and education has received increasing attention as the sustainable development goals deadline of 2030 approaches, yet policy and economic models often address these sectors in isolation. A substantial body of empirical research has demonstrated that health and education are linked through multiple and bidirectional pathways across the life course. An extensive literature has developed exploring how education contributes to better health through improved knowledge, behaviours, staying in school and access to resources; and how good physical and mental health enhances cognitive development, school attendance, and learning outcomes (Cutler and Lleras-Muney 2012). However, only a small number of studies have attempted to quantify the long-term economic benefits of health and education interdependencies through a high-level analysis. The authors are not aware of any studies that have attempted to systematically quantify the bidirectional economic benefits of including health interventions on education outcomes and population health benefits of improved education outcomes using granular, integrated education and economic modelling.

This gap in the evidence base is particularly relevant in the context of current efforts by the United Nations to reform and strengthen the international development and humanitarian system. Initiatives such as UN80, which seeks to improve the effectiveness, integration and impact of UN development activities, and the ongoing Humanitarian Reset, which aims to refocus global humanitarian action on efficiency, coordination and measurable outcomes, highlight the need for more integrated approaches to human capital investment. These reform processes emphasize the importance of cross-sectoral strategies that maximize development impact and ensure that limited resources are directed towards interventions with the strongest social and economic returns. Improved educational and health outcomes can also improve social cohesion and facilitate socioeconomic progress (Camilleri and Camilleri 2016). However, many countries in need of human capital investments are also crisis affected and require humanitarian assistance in addition to human capital investments. For such countries, the measures considered here do not replace such humanitarian assistance but could supplement any such action.

This paper addresses this gap by presenting a conceptual and empirical framework that includes some aspects of the health education relationship and its multi-decade social and economic implications. We use a human capital approach to model how strengthening health interventions aimed at young people (10–24 years)—specifically school nutrition, water, sanitation, and hygiene, deworming and mental health programmes—enhance educational attainment. While interventions aimed at younger cohorts undoubtedly have important impacts on health and education, this study focuses on 10–24-year-olds where barriers to successful completion of school are lack of learning and dropping out. This compares with younger cohorts where the barriers for school include entering school and learning. The age group extends beyond the 10–19-year-old definition of adolescents used by the World Health Organization (WHO 2024a), as up to 20% of young people in low- and middle-income countries (LMICs) do not complete secondary school until their early 20s.

We also examine the impact of improved educational outcomes on under-five and adult mortality rates in subsequent decades, thereby contributing to population health and economic productivity. The study combines an extended version of the Victoria Institute of Strategic Economic Studies (VISES) education model with an employment benefits model (Sheehan et al. 2017) to project returns of health education investments in 75 LMICs from 2025 to 2050. While the health interventions will also lead to health improvements and reductions in disability-adjusted life years (DALYs), the economic impact of these direct health-related benefits is not modelled; only the economic benefits via improvements in educational outcomes.

By integrating education and health benefits into a unified modelling framework, this study contributes to the field of human capital analysis and responds to calls for stronger evidence to guide integrated development strategies. It offers empirical estimates of the benefit-cost ratios (BCRs) of various interventions and quantifies gains in school completions and labour force productivity. It also identifies possible future directions for refining human capital modelling, including incorporating immediate health benefits of interventions, accounting for inequality, social capital, and institutional context.

### Conceptual framework

This paper utilizes a conceptual framework that recognizes and incorporates the bidirectional relationship between education and long-term health impacts of interventions aimed at young people including adolescents. This framework draws upon the importance of these issues for adolescents as described by Ross et al. (2020) and categorized into five domains. The five domains of the adolescent well-being framework include:

- good health and optimum nutrition;
- connectedness, positive values and contributions to society;
- safety and a supportive environment;
- learning, competence, education, skills and employability; and
- agency and resilience. (WHO 2024b)

The bidirectional relationship between education and health addresses several of these domains including good health and optimum nutrition, learning competence education, skills and employability, as well as agency and resilience.

#### Human capital framework for health and education

Human capital is ‘the skills the labour force possesses’ and is regarded as a resource or asset. It encompasses the notion that there are investments in people (e.g. education, training and health) and that these investments increase an individual’s productivity (Goldin 2024).

The inclusion of human capital in economic growth accounting considers increases in education as enhancing the productivity of individuals. Differential productivity is measured by how much higher earnings are for workers with different levels of education (Goldin 2024).

Since Mincer (1958), Schultz (1961) and Becker (1962) published their studies on human capital, a very large evidence base has established that both education and health are core components of human capital (see e.g. Jamison et al. 2005, Orazem et al. 2022, Ridhwan et al. 2022, Chang et al. 2024). More and better education will increase growth rates of national income (Jamison et al. 2007), while healthier individuals and populations are likely to generate higher economic output than unhealthy ones and live longer (Schäferhoff et al. 2016, Pradhan et al. 2017). Individuals with better physical and mental health are more productive at work and less likely to be absent from work. Healthier children are more likely to go to school, to learn, and to develop to their full human and economic potential (Well 2007, Ogbuoji et al. 2020, Norwich et al. 2022, WHO 2024b). Their interdependence has large implications for economic development, social mobility and cohesion, and intergenerational equity (Card et al. 2018). These links emphasize the importance of health in educational settings in human capital development (Cohee et al. 2020). Consequently, the human capital framework provides a useful framework to undertake economic modelling of the links between education and health (Goldin 2024).

#### Education as investment in human capital

Education is a vital investment in human capital, enhancing individuals’ skills, knowledge and productivity. By improving cognitive abilities and fostering critical thinking, education increases a person’s potential to contribute to economic growth and innovation. It also leads to higher lifetime earnings, better employment opportunities. and improved health outcomes. For societies, investing in education for people in all wealth brackets reduces inequality, boosts social mobility, and strengthens civic participation with the reverse also true with unequal access risking social corrosion and conflict (Ng 2017). Those with more schooling are more likely to understand health information, access healthcare and make healthy choices (Contoyannis and Jones 2004). The benefits extend across generations, as educated individuals are more likely to support the education of their children (Abraham and Mallatt 2022). While education can include many different variants, only formal school education is considered in the modelling.

#### Health as investment in human capital

Health improvements are a key investment in human capital, directly enhancing individuals’ ability to learn, work and contribute productively to society (WHO 2024c). Good physical and mental health boosts physical and cognitive functioning, reduces absenteeism, and increases the capacity to benefit from education and training (Norwich et al. 2022, Stephens and Toohey 2022). Healthier populations tend to have longer working lives and lower healthcare costs, supporting economic growth and social stability. Early life health interventions (below the age of 10), yield long-term returns by improving educational attainment and future earnings, as well as health interventions through later years into adolescence. Numerous studies show physical health affects earnings, even when education is controlled for (Suhrcke et al. 2011, Stephens and Toohey 2022).

### Adolescent well-being definition and conceptual framework

In 2020, the UN H6+ published a definition and conceptual framework for adolescent well-being, consisting of five interconnected domains (Ross et al. 2020). The definition is: ‘Adolescents have the support, confidence, and resources to thrive in contexts of secure and healthy relationships, realizing their full potential and rights’. The five domains of adolescent well-being are: good health and optimum nutrition; connectedness, positive values, and contribution to society; safety and a supportive environment; learning, competence, education, skills, and employability; and agency and resilience. A key aspect of the framework is the interconnectedness of the domains, and how achievement of one supports the others. This paper aims to build on the framework to demonstrate the interconnectedness of the domains of good health and optimum nutrition, and learning, competence, education, skills, and employability.

### Links between health and education

A growing body of research has examined the interaction between education and health that reveals a consistent pattern: individuals with lower levels of education tend to experience poorer health outcomes. This is evident even in high-income countries (HICs) such as the United States (Raghupathi and Raghupathi 2020) and the United Kingdom (Liu et al. 2024a). This is considered to be largely due to the health disparities shaped by educational attainment. While this pattern is clear, a significant challenge is the complex interplay between education and health, and how it changes over the life course and across generations, thereby making it difficult to determine causal pathways. This association is embedded within broader social, economic and policy contexts, further complicating efforts to fully understand the mechanisms at play (Zajacova and Lawrence 2018).

#### Education’s impact on health

The potential causal pathways through which education affects health identified in the literature are numerous and interconnected. First, considerable evidence suggests educational systems and curricula evolve over time, altering the knowledge and skills imparted to each generation, which subsequently has a varying influence on health through time and the life course (Lynch 2003, Raghupathi and Raghupathi 2020). Second, the relationship between variables—such as income, occupation, or workplace conditions—and health outcomes is not constant. As healthcare costs rise or as industries become safer or more hazardous, the way education influences health may shift. Third, some studies indicate that relative changes in socioeconomic status from increased educational attainment can significantly impact health (Carrilero et al. 2021). Also, changes in population-level health and mortality patterns suggest that the risks and pathways to ill health have evolved, which may, in turn, affect how education shapes health trends (Raghupathi and Raghupathi 2020).

Studies have identified three broad categories of causal pathways that link education to health: economic, social and psychological, and behavioural (Ross and Wu 1995). Economically, higher levels of education often lead to better jobs and higher incomes, which in turn facilitate access to quality healthcare—both preventive and curative (Landerman et al. 1994, Andersen 1995, Ukert et al. 2022). Social and psychological factors, including problem-solving abilities, resilience, distress and anxiety, social support and emotional regulation, also impact the relationship between education and health (Wheaton 1983, Manton et al. 1997, Browning et al. 2015, Ocloo et al. 2021, Khatri and Assefa 2022, Chang 2025). These traits help individuals cope with stress and illness more effectively (Harper et al. 1994). Better-educated individuals typically report higher levels of overall health, and experience lower rates of illness, disability and premature death (Case and Deaton 2017). Conversely, low educational attainment is associated with worse health indicators, such as lower life expectancy and greater vulnerability to disease (Hardarson et al. 2001, Krueger et al. 2015). A range of factors contribute to this relationship, including early life health conditions, family background, access to health information, and broader social networks (Cutler and Lleras-Muney 2006, 2012).

Evidence also shows a strong link between the amount and quality of formal education and the uptake of preventive healthcare service, as well as reducing the prevalence of depression (Folland et al. 2024). As such, at a societal level, education is increasingly recognized as a critical social determinant of health (Zimmerman and Woolf 2014). Education builds a range of human capital attributes—such as cognitive skills, self-efficacy, and decision-making ability—that directly and indirectly contribute to improved health (Mirowsky and Ross 2005). Over time, education has contributed to economic growth, job stability and social progress (Baker 2014). Countries that implement policies to expand access to and improve the quality of education often realize long-term public health benefits, including reductions in smoking, obesity, and communicable and non-communicable preventable diseases (Baker 2014). While education is universally beneficial, the impact and specific approaches may vary across regions due to differences in epidemiological and cultural contexts, existing health systems and socioeconomic factors (Liu et al. 2024b).

Consequently, education systems influence health as higher educational attainment correlates with better self-rated health and greater disease awareness (Baker 2014). Importantly, the relationship between education and health is bidirectional. Poor educational outcomes are often linked to poor health, as limited resources, poor nutrition, or unsafe neighbourhoods can create barriers to learning. Conversely, health issues in early life, such as chronic illness or cognitive impairments, can hinder educational attainment. This cycle reinforces disadvantage across generations and highlights the need for integrated policy approaches (Ro et al. 2016). This is discussed in more detail in the following section.

#### Health’s impact on education

Health and health systems play a fundamental role in shaping subsequent outcomes, including educational outcomes such as years of schooling, secondary school completions, and academic achievement (Currie et al. 2010, Almond and Currie 2011, Currie and Vogl 2013). This impact begins at an early age, as being born with low birthweight and lacking access to appropriate neonatal healthcare significantly raises the risk of illnesses and diseases that can hinder cognitive development and learning ability (Alcaraz et al. 2017).

Direct effects on education occur when health conditions directly impact learning. For example, the ‘foetal origins hypothesis’ suggests that poor prenatal environments can permanently impair bodily and brain functions (Almond and Currie 2011). Also in infancy, low birthweight and stunting can delay cognitive development, immature brain structures, or birth complications (Hintz et al. 2005). In childhood and adolescence, illness or malnutrition can impair concentration and development (Bryan et al. 2004, Costello et al. 2021). Poor physical and mental health can lead to school absences (Rafiq et al. 2022) or shift spending away from education by the family of the student (Cutler and Lleras-Muney 2012), as well as difficulties coping with the school and social environment (Rafiq et al. 2022). At each life stage, health affects education through both direct and indirect mechanisms, as demonstrated conceptually in Fig. 1.

**Figure 1:**
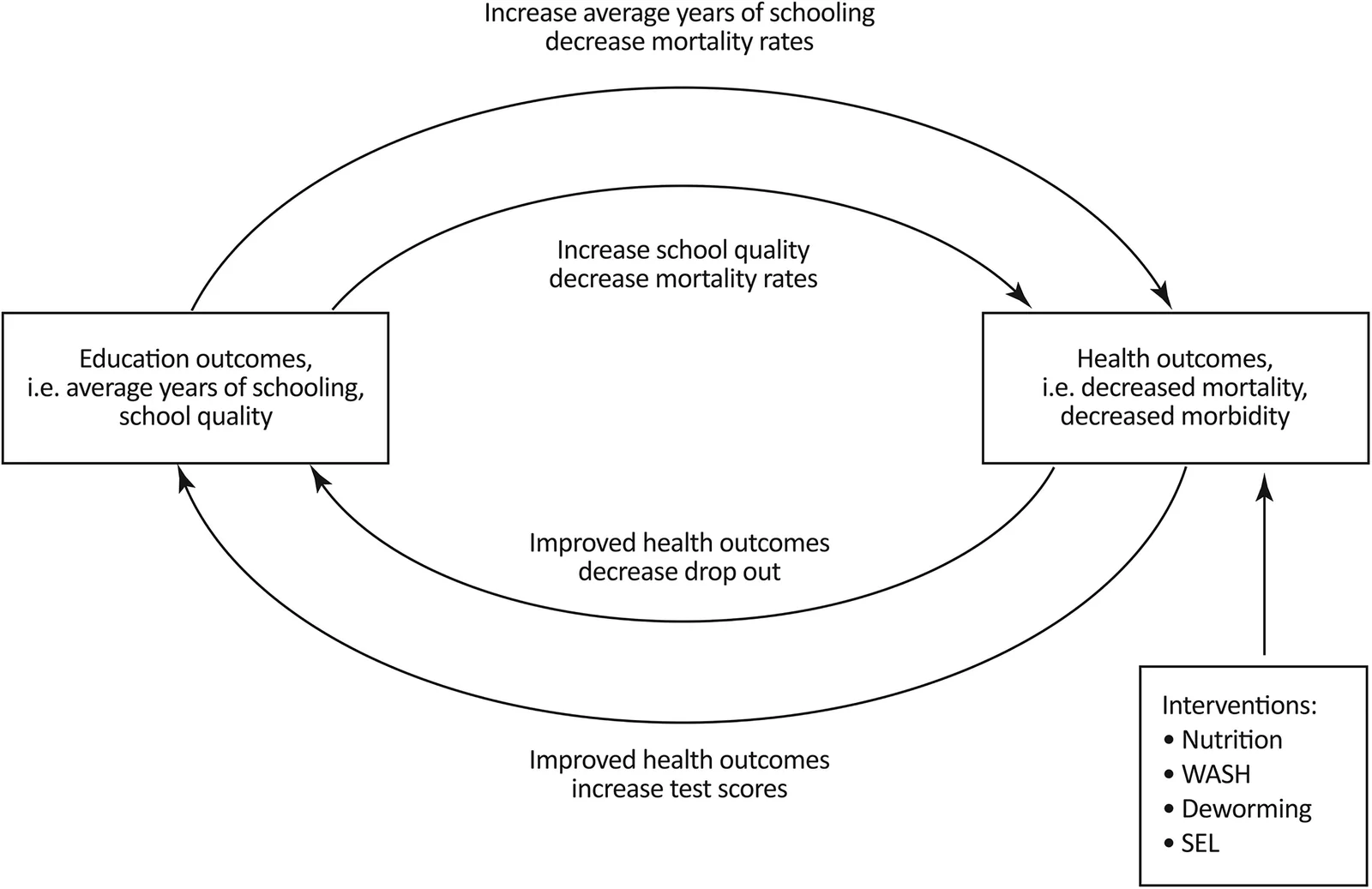
Health impact on education.

While health interventions bring an immediate health benefit to students who are included in any intervention, including mental health, these would be expected to reduce DALYs as well as infant mortalities (Tung et al. 2023). This reduction is not calculated in this study, but rather the long-term decreased mortality rate impact of improved education is used. In addition, parent training has been shown to reduce infant mortality. The estimation of the economic benefits of these immediate health improvements are a possible extension to this study.

It is important to note that while the impact of health-education varies across the life course, it is a consistent feature for members of the community with disabilities. Living with disabilities can lead to greater challenges for their access to quality health services that can also impact their education, and consequently, their human capital development. However, while this is an important issue, consideration of specific interventions addressing these challenges is beyond the scope of the study.

## Methods

This section outlines the methodology for identifying and modelling health-related interventions with demonstrated potential to improve educational outcomes as well as discussing the benefits of education on health. Building on evidence from numerous meta-analyses and systematic reviews, four health-focused education intervention types were selected for detailed analysis: school meals/nutrition, deworming, water, sanitation, and hygiene (WASH), and mental health/social-emotional learning programmes. These interventions were chosen from numerous studies outlining effectiveness and cost base for their strong empirical links to reduced dropout rates and improved test scores, as summarized in Table 1. Subsequent subsections describe each intervention and its measured educational impacts, which form the basis for integrating health effects into the education-health economic model presented in section ‘Economic modelling of education-health links’. While a category of interventions have many similar features, due to the wide variation in factors such as implementation settings, cohorts, gender and details of particular interventions in more than 70 different implementation countries, the interventions modelled are categories of interventions as opposed to specific interventions and the effectiveness assigned to them are averages from numerous studies.

**Table 1:** Education benefits of modelled health interventions (average values calculated from meta-analyses and literature reviews referred to in study).

Health interventions	Average dropout reduction	Average test score improvement
Nutrition	9.5%	3%
Deworming	5%	20%
WASH	18%	0%
Mental health programmes, e.g. SEL	5%	25%

WASH, water, sanitation and hygiene; SEL, social and emotional learning.

Source: Authors’ calculations.

### Health interventions for improved education outcomes

To illustrate the education impacts of improved health, specific health-related interventions must be identified that demonstrate the capacity to improve education outcomes. There have been numerous meta-analyses and systematic reviews that examine a large range of interventions to improve educational outcomes. The reviews and meta-analyses examined for this study were Angrist et al. (2020), Masino and Niño-Zarazúa (2016), Damon et al. (2019), Springer and Miller-Grandvaux (2022), Asim et al. (2015), Ganimian and Murnane (2016), Sampa et al. (2021), Evans and Popova (2016), Evans et al. (2024) and Conn (2017), with specific examples including the School Medical Inspection initiative in Sri Lanka (Wickramasinghe et al. 2019).

The interventions examined for potential inclusion range from improved pedagogy, teaching at the right level, pupil–teacher ratio, improved materials, cash transfers, proximity of schools, and many others including several that impact on student health. While non-health-based interventions are often considered the most cost effective, the reported studies do not consider the bidirectional health benefits. As the focus of this study is to examine the bidirectional health and education benefits, the four most effective health-related types of interventions identified in the 10 meta-analyses and reviews have been included in the quantitative analysis. The interventions selected for the model include providing nutritional supplements or school meals, deworming, and improving WASH, as well as mental health programmes. The average impacts of these four types of interventions on educational dropout and test scores reported in the reviews and meta-analyses mentioned above are summarized in Table 1 and discussed in more detail in the following sections. Interventions related to myopia were not included, because some of the parameters required by the current model were not available in the literature.

#### Meals/nutrition

Numerous studies have shown that improved nutrition or school meals can lead to better educational outcomes (Glewwe and Miguel 2007, Jomaa et al. 2011, Pongutta et al. 2022). Consequently, a common intervention is to provide school meals (or take-home supplies) or supplements. Studies of school meals/nutrition supplements interventions have varied both in the specific education-related outcomes measured and in the effect sizes they found depending on the programme (Whaley et al. 2003). For example, in Guatemala, a protein-rich supplement raised grades and reading scores, especially for girls (Maluccio et al. 2009). Iron supplements in China also improved test scores (Luo et al. 2012). Breakfast programmes show promise: Norway’s ‘Oslo breakfast’ increased the amount of schooling by an average of 0.1 years (Bütikofer et al. 2019), while U.S. programmes enhanced mathematics results (Frisvold 2015). In the studies that reported these two outcomes, the average impact on educational dropout and test scores was a reduction of 9.5% and improvement of 3%, respectively (Table 1).

#### Water, sanitation, and hygiene

Numerous studies have found that improving clean water access, sanitation (including menstrual care for girls) and hygiene infrastructure or providing latrines to improve sanitation, improves inclusiveness and student learning and reduces dropout rates (Jasper et al. 2012, McMichael 2019). In Kenya, a programme saw 58% fewer missed days (Freeman et al. 2012), while a programme in Egypt observed 21% fewer absences (Talaat et al. 2011). While WASH reduces dropout rates, there is a lack of evidence in the literature with respect to its impact on test scores. Consequently, WASH is modelled as having no impact on test scores.

#### Deworming

Deworming programmes have been shown to reduce school dropout rates, particularly in low-income regions. Parasites can cause anaemia, fatigue and cognitive impairments, which hinder academic performance and attendance (Taylor-Robinson et al. 2015). Deworming programmes can effectively reduce the burden of intestinal worms, and improving children’s nutritional status and energy levels, which boosts focus and participation in school (Ali et al. 2019). Healthier students are more likely to stay in school and perform better academically. These interventions contribute to breaking the cycle of poverty by enabling consistent education and better long-term opportunities (Ahuja et al. 2017).

#### School health and well-being programmes

Social and emotional learning (SEL) programmes broadly encompass health and wellness initiatives in schools. These programmes vary widely, with each tailored to meet the specific goals and context of individual schools. While most SEL programmes include elements that promote behavioural skills and support mental health, the balance between these components differs. Some programmes primarily target the reduction of depression and anxiety, whereas others focus on mitigating delinquency, addressing conduct disorders, or preventing substance abuse. For example, initiatives like responsive classroom are designed to provide teachers with strategies, practices and techniques to improve their efficacy to impact student social and emotional, academic, and other non-academic outcomes (Belfield et al. 2015). This diversity in design and objectives can complicate the evaluation of SEL programmes and lead to inconsistent results.

Social and emotional skills are increasingly recognized as key predictors of success in education, employment and broader life outcomes (Heckman and Kautz 2012). SEL is often integrated into school curricula to help reduce risky behaviours, such as violence and substance use (Durlak et al. 2011). It also plays a vital role in enhancing non-cognitive skills and boosting academic performance (Gutman and Schoon 2013).

Further supporting this connection, Walberg et al. (2004) examined the influence of SEL on various educational outcomes, including students’ attitudes (such as motivation and responsibility), behaviours (like attendance and study habits), and performance (grades and test results). A review of 80 nationally available programmes revealed that 83% led to measurable academic gains (CASEL 2003).

### Economic modelling of education-health links

This section explores the economic modelling of the link between education and health, focusing on how improvements in health can influence educational outcomes, as well as the converse where improved education leads to improved health outcomes, specifically reduced mortality rates. The modelling of this bidirectional relationship draws upon human capital theory highlighting the mechanisms through which health acts as both an input to and an outcome of education. Integrating health into an economic model of education enables better understanding of the long-term returns to health and education interventions.

#### Education-health modelling approach

The modelling approach taken for this study incorporates the bidirectional links between health and education as discussed in sections ‘Methods’ and ‘Results’ through the following approaches.

- The effect of health and its impact on education. As discussed in the previous section, selected health interventions substantially increase time in school and academic achievement.
- The effect of education (quantity and quality) and its impact on health, specifically quantified as:
  ○ reduced adult rates of mortality; and
  ○ reduced under-five mortality.

The bidirectional links for the model are illustrated in Fig. 2.

**Figure 2:**
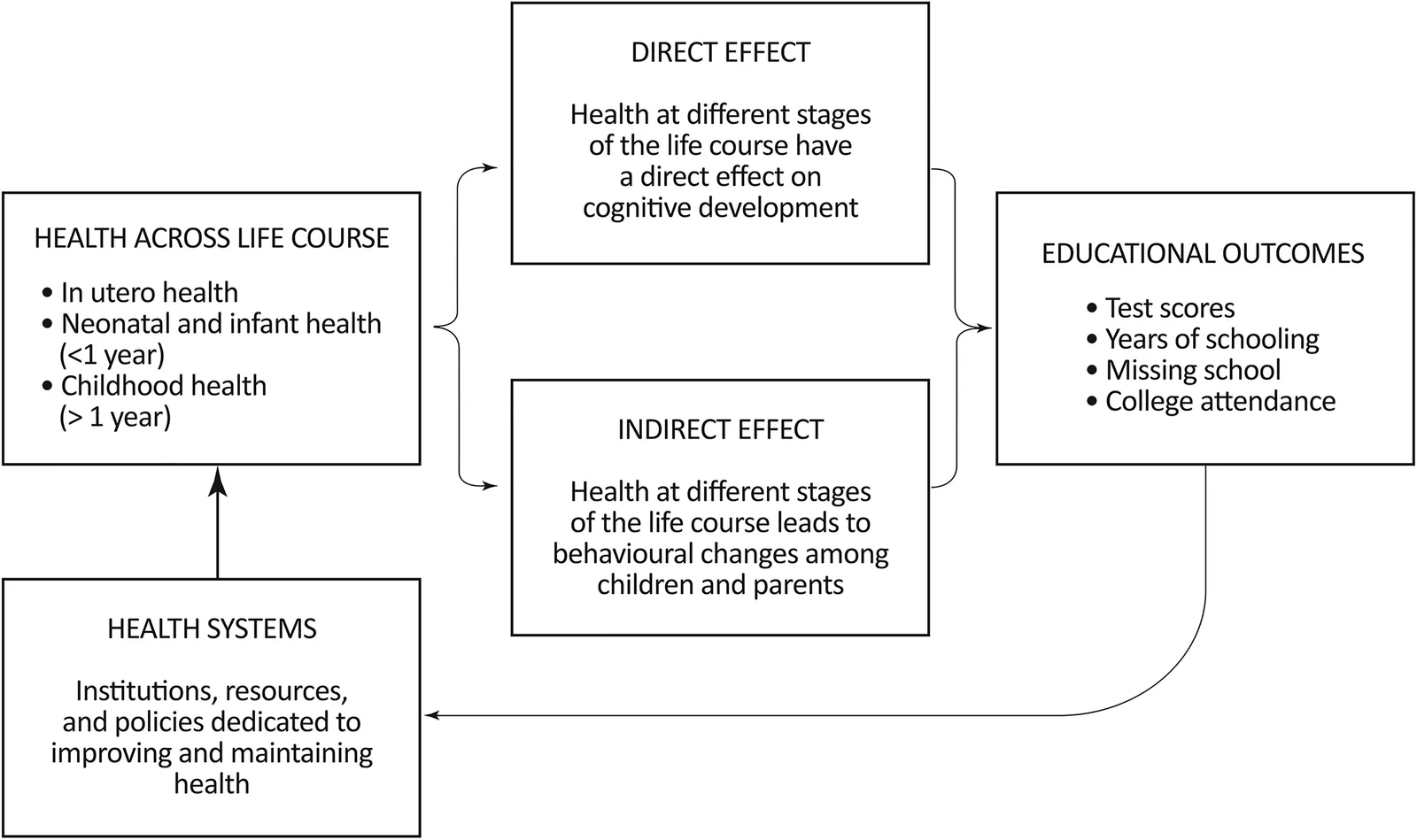
Health-education links model structure.

The two-way linkages are incorporated into an extended version of the pre-existing VISES education model through several mechanisms. This version of the VISES education model is an evolution of the model described in Wils et al. (2019), and subsequently developed further in succeeding projects for the World Health Organization (Sheehan et al. 2022), UNICEF (Rasmussen et al. 2022) and the Partnership for Maternal, Newborn, and Child Health (WHO 2024b). Firstly, improved health leading to better education outcomes is an existing feature of previous versions of the model and these are included in the modelling. These interventions have previously been modelled in conjunction with non-health-related interventions, such as improved pedagogy and conditional cash transfers. However, for this study, only interventions with an explicit health focus have been included to isolate the educational impacts of improved health. The economic benefits of immediate health improvements are not a feature of the current version of the model, though future versions of the model aim to include this.

##### Modelling health impacts of interventions to improve education outcomes

The modelling approach for the health benefits of education is considered through decreased mortality rates derived from the Schäferhoff et al. (2016) study on the health benefits of education. Schäferhoff et al. (2016) found an association between lower mortality rates and increased average years of schooling. Specifically, they found:

- A 1-year increase in mean years of female schooling in LMICs between 1970 and 2010 was associated with reductions in female and male adult mortality of 3.7% and 2.2%, respectively; and was also associated with a decline in under-five mortality by 4.2%.
- School quality, measured as international test scores (Wils et al. 2019), also has a substantial effect on health outcomes. As data from LMICs was limited, Schäferhoff et al. (2016) used data from HICs and found that a one standard deviation improvement in school quality (as measured by test results) was associated with a 2.4% lower overall adult mortality rate, a 2.3% lower adult female mortality rate, and a 3.4% lower under-five mortality rate. This result was applied generally to countries from all income levels.

Many of the data on the effectiveness of interventions that were used as inputs to the models in this study were based on the results of observational studies, with the assumption made that the associations reported were causal. The values of the health benefits of increased quantity and quality of education are summarized in Table 2.

**Table 2:** Lower mortality rates associated with improved education-related outcomes.

Education outcome	Both	Male	Female
	0–4 years	15–59 years	15–59 years
Increase of 1 yr in average years of female schooling	4.2%	2.2%	3.7%
Increase of 1 SD in average test scores	3.4%	2.5%	2.3%

SD, standard deviation.

Source: Schäferhoff et al. (2016).

The extended VISES education model used here is a multi-country education aggregation model that projects education outcomes out to 2050.

Three major hurdles exist to completing primary and secondary school: entering school, remaining in school and learning in school. The first two of these closely correspond to pre-adolescence and adolescence, respectively. As such, the education challenges in pre-adolescence and in adolescence are quite distinct. For children under 10, education barriers relate mostly to entry; for adolescents, the risks relate to drop out before completing secondary school. This study, which is focused on education risk for young people aged 10–24, concentrates on dropout risks, as well as learning that applies to both pre-adolescence and adolescence (Wils et al. 2019).

The model calculates school completions with a matrix of pupils by age, grade, and gender over time. The projections depend on the initial intake of students, flows to a next grade based on promotion, dropout or transition rates, and to the same grade in the next year based on repetition and dropout rates. These rates are based on historical trends to generate base case secondary school completions, as well as years of schooling completed for each cohort and average years of primary and secondary school. The model overlays on top of the base case projections with an intervention module that changes education outcomes. The values for coverage and effectiveness of the interventions’ impacts on drop out, promotion, repetition and learning changes from the base case data to the values from the interventions. In this version of the model, only health-based interventions are included that lead to improvements in these outcomes. The cost of the interventions consists of the cost of the interventions themselves, and the cost of educating the additional number of students attending through to the final year of secondary school due to the interventions.

The health impact of education is incorporated into the education-health model by adjusting population projections from a reduction in under-five mortality rates due to increased average years of schooling and school quality. This under-five mortality reduction applies after the first cohort of students has passed through secondary school having experienced the interventions for all their years in secondary school. The reduced impact on adult mortality is not applicable to the education model, as the education model only considers the population up to the age of 24 years. Consequently, the adult mortality benefits are incorporated into the employment benefits model that utilizes the educational outcomes from the VISES education model described below.

#### Employment benefits modelling approach

The employment benefits model outlined here is based on the model developed and described in detail by Sheehan and Shi (2019) and extended in subsequent projects for the WHO (Sheehan et al. 2022) and UNICEF (Rasmussen et al. 2022). The model focuses on two distinct effects of improved education outcomes on employment. The first is how increased years of schooling and better-quality education enhance an individual’s productivity and earnings in a given job, drawing on extensive research on returns to schooling. The second is education’s role in enabling young people to access better-quality employment, especially relevant given the dominance of low-wage informal work in LMICs. These two effects—productivity within jobs and access to a wider range of jobs—are usually treated separately in development research, with limited connection between them. However, both are crucial in improving national productivity and economic growth. A central finding from a large body of literature, despite some critiques, is that improving educational outcomes—both in terms of completed years of formal education and learning quality judged by performance in standardized tests—yields substantial returns in gross domestic product (GDP) and GDP growth (Hanushek and Woessman 2015).

However, these two dimensions (school quantity and quality) do not consider the actual employment structure in many LMICs where most employment is usually in the informal sector, whereas most research has focused exclusively on formal jobs.

In most LMICs, the proportion of the working age population who lack any formal employment may be quite low, however, underemployment (only working part-time) and informal work is very common. While a substantial proportion of youth are often officially recorded as being not in education, employment, and training, some of them may actually be in part-time or full-time informal work and/or training with education levels playing a key role in shaping these labour force patterns.

Consequently, the model of the impact of better secondary education on employment and productivity outlined here includes four channels through which better education influences these variables, relative to an unchanged policy base case (Sheehan and Shi 2019):

1. Increased years of schooling lead to higher productivity and higher earnings in employment.
2. Improvements in the quality of education (including teaching standards, materials, etc.), also lead to higher productivity and earnings in employment.
3. A rise in secondary school completions leads to an increase in the relative share of formal (usually higher-paid) employment and a decline in the share of informal employment.
4. An increase in secondary completions also leads to a rise in the proportion of the population who are employed.

In addition to these channels, an additional channel of benefit is the decrease in adult mortality rates through more and better schooling as identified by Schäferhoff et al. (2016) that leads to increased population projections relative to the base case and applied annually to the cohorts that have passed through and exited secondary school, whether completed or not, in the model. That increased population having better education outcomes from additional years of schooling and commensurate higher levels of GDP as described by Sheehan and Shi (2019).

#### Education Health model and employment model combination

The combination of the two models starts with estimates, relative to an unchanged policy base case, of the improved educational outcomes obtained through the education-health model from a set of health-based interventions to reduce school dropout rates and improve school quality. Three sets of results for females and males aged 10–24 years, namely years of completed schooling, average school quality, and the proportion of the population that has completed secondary school are inputted into the employment benefits model. The modelling included cohorts up to the age of 24 as in some countries up to 20% of young people do not finish their formal education until their 20s. The application also uses an estimate of the population of 20- to 24-year-olds who are not in school and an exogenous estimate of GDP per capita for each country and year. Each successive 10- to 24-year-old cohort is traced across time as they age and become an increasing share of the national labour force to estimate the growing impact of better education on national productivity, employment and GDP over time.

## Results

The model was run for 10- to 24-year-olds in 75 LMICs with results calculated for the health interventions with education benefits only, and then with the health benefits only (reduced mortality), as well to illustrate the impact of including the health benefits of education.

### Education and economic results

The initial results with only the education benefits of health interventions is consistent with previous modelling (Rasmussen et al. 2022, Sheehan et al. 2022, WHO 2024b), where health benefits (reductions in mortality rates) were not included in the estimates. The second set of results also compares to a business-as-usual base case, but includes the mortality rate reductions from increased quantity and quality of education. The weighted results refer to the sum of benefits and costs for all countries, while the unweighted results refer to the average of all the individual country’s results.

The results of the modelling indicate the importance of both the educational benefits of health interventions, as well as the health benefits of increased quantity and quality of education (Table 3). The increase in school completions is important for the health interventions on their own, leading to an ∼9.1% increase in school completions (from 4635.3 million to 5058.1 million) and economic benefits of over US$15 283 billion at a cost of US$2193 billion resulting in an unweighted (the sum of benefits of all countries divided by the sum of costs of all countries) BCR of 6.97. The average of each individual country’s BCRs is 10.65 indicating that several smaller countries have very high BCRs leading to a higher average figure.

**Table 3:** Education and economic outcomes from modelled interventions.

Scenario	Base case school completions 2025–2050 (mil)	Intervention school completions 2025–2050 (mil)	Additional school completions 2025–2050 (mil)	Benefits (NPV) 2025–2050 (US$ mil)	Costs (NPV) 2025–2050 (US$ mil)	Weighted BCR	Average unweighted BCR
Health interventions education benefits only	4 635.3	5 058.1	422.8	15 282 725	2 193 174	6.97	10.65
Both education and health benefits	4 635.3	5 063.9	428.7	25 212 846	2 199 741	11.46	20.17

NPV, net present value; BCR, benefit-cost ratio.

Source: Authors’ calculations.

The same substantial results are true for the alternate scenario where both educational benefits and health benefits are included; however, this scenario has a slightly higher number of school completions with a 9.2% increase (Table 3). A relatively small difference is expected in school completions as the improved educational outcomes lead to a small decrease in the 0–5 mortality rates that consequently leads to a small increase in the number of school completions. However, by far the more substantial impact is on the overall economic benefits that arise from inclusion of the reduced mortality rates for adults, as these are included in the employment model, rather than a 5-year cohort of population projections included in the education model. While a similar number of young people complete school, a larger number of these people survive through to age 65 leading to significant economic benefits. This significantly higher result is expected as the reduction in adult mortality applies to an age cohort 10 times larger (aged 15–65) than the under-five mortality reduction, and hence has a much larger economic impact due to increased survival and corresponding contribution to GDP. The results are economic benefits of US$25 213 billion at a cost of US$2200 billion leading to a BCR of 11.46 for the combined results of all countries and an unweighted average BCR value of 20.17 (Table 4).

**Table 4:** Adult mortality reduction, costs and benefits.

Costs	US$2200 billion
Benefits	US$25 123 billion
Weighted benefit-cost ratio	11.46
Unweighted benefit-cost ratio	20.17

Source: Authors’ calculations.

## Discussion

The results of this study give further evidence to the bidirectional relationship between two of the five domains in the adolescent well-being framework (Ross et al. 2020); good health and optimum nutrition, and learning, competence, education, skills, and employability. A key aspect of the framework being the interconnectedness of the domains and how success of one supports the others, with the results of this study supporting this contention with substantial long-term economic benefits of integrating health interventions with education interventions in schools.

Drawing on a human capital framework and using an extended education and economic modelling approach, we find evidence that investments in adolescent health through the most effective interventions identified in numerous meta-analyses and reviews generate long-term improvements in educational attainment, while higher levels of education significantly improve population health outcomes and commensurate economic gains.

This study examines the 10- to 24-year-old cohort that extends beyond adolescence as defined by the WHO as being 10–19 years old (WHO 2024a), as many young people in LMICs do not finish secondary school until their early twenties which is found in education attainment from household surveys (Demographic and Health Survey and Multiple Indicator Cluster Survey). As discussed in section 3, to exclude this cohort would fail to capture a significant section of the population and the associated benefits.

The modelling results highlight the scale of potential gains. Health interventions that improve educational outcomes—through nutrition, WASH, deworming, and mental health—produce over 422 million additional school completions between 2025 and 2050 across 75 LMICs. These gains translate into economic benefits exceeding US$15.3 trillion, with a weighted BCR of 6.97. When the mortality reductions associated with improved education are also included, the benefits rise to over US$25.2 trillion, with a weighted BCR of 11.46. These figures underscore the high returns to health-related interventions that are traditionally not classified as part of the education system, but directly affect students’ learning capacities and outcomes.

The inclusion of mortality benefits from increased average years of schooling and improved school quality represents an important methodological advance in the VISES education and employment models. By capturing both education’s effects on under-five and adult mortality and health’s influence on educational attainment, the model provides insight into the long-term and intergenerational effects of human capital investments in adolescents in health and education. The findings show that the most substantial economic benefits are generated not only through additional years of schooling, but also through the survival of a greater proportion of the population to working age, thus enlarging the productive labour force. The inclusion of health-related interventions achieve the double benefit of both improving both health and education outcomes, that then also further improve health outcomes (Abdul et al. 2024). Such benefits come from the modelled health-related interventions. However, such results are illustrative as opposed to definitive, as other, health-related interventions yet to be documented in terms of cost effectiveness, could also have beneficial impacts both on health and education outcomes.

This study confirms the substantial and synergistic economic returns to coordinated investments in health and education. It provides a strong empirical foundation for integrated, multi-sectoral policy action and identifies important directions for extending current modelling approaches to better reflect the full spectrum of human capital development. The results of this study are therefore highly relevant to inclusive policy and planning.

### Limitations

Despite these significant results, the current modelling framework has limitations, as it does not estimate the economic gains from immediate health interventions. These benefits are likely to be significant with cursory estimates of potentially reductions in millions of DALYs per annum. The modelling framework is also grounded in a conventional human capital approach that has limitations, as it primarily focuses on market-based outcomes, such as earnings and GDP growth. The limitation of the conventional human capital approach is increasingly being examined through initiatives such as the Beyond GDP initiative (UN 2023). This approach potentially underestimates important and likely substantial non-economic and social returns. For example, improved wealth and gender equality, and enhanced civic engagement are critical benefits of health and education investments in schools that are not fully captured in the current modelling. Furthermore, the model assumes uniform intervention effects across countries and populations, which may mask the significant role of contextual factors such as social capital, institutional quality, and wealth and geographic inequality that also impact on social cohesion.

To address these limitations, future research could expand the modelling framework to estimate the immediate economic benefits of health improvements, as well as incorporate broader determinants of health and education outcomes. This may include the role of different contexts such as institutions, community and family, wealth inequality, and social cohesion together with social capital. To address these limitations, the current modelling approach could potentially be broadened to include inequality in institutions, communities and families, as well as geographic and social cohesion and social capital inequalities.

The additions listed above would strengthen the model’s relevance for inclusive policy design and could be explored using recent case studies undertaken in this area by the authors in Colombia, South Africa, and India (VISES 2024).

## Conclusion

This study provides solid evidence that the health- and education-related benefits from health and education interventions in schools are mutually reinforcing and generate high economic returns. Health interventions that improve educational outcomes—including interventions to improve nutrition, deworming, WASH, and mental health—result in significant increases in school completions and long-term earnings. When paired with the health benefits of increased education, including reductions in child and adult mortality, the models suggest that the compounded benefits would exceed US$25.2 trillion between 2025 and 2050 across 75 LMICs, with the estimated weighted average BCR being 11.46. These results offer a compelling case for targeting health and education investments towards where the marginal returns are highest.

The framework primarily captures market-based outcomes and assumes relatively uniform effects across contexts. Future research should incorporate the economic benefits of immediate health improvements, broader human development outcomes, such as reduced inequality and social capital formation, and include institutional, community and geographic variations.

Overall, this study underscores the value of holistic, evidence-based investment in human capital and offers a foundation for more inclusive and impactful development strategies in the coming decades.

## Data Availability

The data underlying this article are available from the World Bank Data Bank, UNESCO Data repository, UN World Population Prospects, the DHS Program, UNICEF Multiple Indicator Cluster Surveys. The datasets were derived from sources in the public domain: https://data.worldbank.org/, https://www.uis.unesco.org/en/data, https://population.un.org/wpp/, https://www.dhsprogram.com/, https://mics.unicef.org/.

## Reflexivity statement

The research originated from a global study into the urgent need for investment in adolescent well-being including health and education especially in low- and middle-income countries. The authors include four women and five men with professional experience across multiple levels and countries. All authors specialize in various areas of public health and health policy. This study has benefitted greatly from the considerable expertise of the authors across the globe and their work across varied geographies within public health practice, economic modelling and analysis, research and policy making.
